# Three Ports Laparoscopic Resection for Colorectal Cancer: A Step on Refining of Reduced Port Surgery

**DOI:** 10.1155/2014/781549

**Published:** 2014-03-12

**Authors:** Anwar Tawfik Amin, Tarek M. Elsaba, Gamal Amira

**Affiliations:** ^1^Department of Surgical Oncology, South Egypt Cancer Institute, Assiut University, Assiut, Egypt; ^2^Department of Pathology, South Egypt Cancer Institute, Assiut University, Assiut, Egypt; ^3^Department of Surgical Oncology, National Cancer Institute, Cairo University, Cairo, Egypt

## Abstract

*Background*. Reduced port surgery (RPS) is becoming increasingly popular for some surgeries. However, the application of RPS to the field of colectomy is still underdeveloped. *Patients and Methods*. In this series, we evaluated the outcome of laparoscopic colorectal resection using 3 ports technique (10 mm umbilical port plus another two ports of either 5 or 10 mm) for twenty-four cases of colorectal cancer as a step for refining of RPS. *Results*. The mean estimated blood loss was 70 mL (40–90 mL). No major intraoperative complications have been encountered. The mean time for passing flatus after surgery was 36 hours (12–48 hrs). The mean time for oral fluid intake was 36 hours and for semisolid food was 48 hours. The mean hospital stay was 5 days (4–7 days). The perioperative period passed without events. All cases had free surgical margins. The mean number of retrieved lymph nodes was 14 lymph nodes (5–23). *Conclusion*. Three ports laparoscopy assisted colorectal surgeries looks to be safe, effective and has cosmetic advantages. The procedure could maintain the oncologic principles of cancer surgery. It's a step on the way of refining of reduced port surgery.

## 1. Introduction

Laparoscopy has emerged as a useful tool in the surgical treatment of the colon and rectal diseases. Specifically for colon cancer, a laparoscopic approach offers short-term benefits to patients while it looks to maintain long-term oncologic outcomes. Favorable postoperative results in terms of less pain, less consumption of analgesia, early return of bowel function, and short hospital stay in patients who underwent laparoscopic colorectal surgery have been persistently reported, both in series with benign and malignant colorectal diseases [1–4]. Published randomized trials comparing laparoscopic and open colorectal resection did not show inferior oncologic results in patients who underwent laparoscopic surgery [5–9].

The added advantages of improved morbidity and cosmesis after laparoscopic surgery make reduction of ports number or even single incision laparoscopic colectomy a viable alternative to the conventional multiports laparoscopic colectomy. However, reduced ports surgery (RPS) as well as single incision laparoscopic colectomy (SILC) are challenging and highly demanding techniques.

In this series, we have evaluated the outcome of our newly developed technique of laparoscopic resection of colorectal cancer, only using three ports as a step on refining of reduced port surgery.

## 2. Patients and Methods

Twenty-four patients (13 males and 11 females) have been enrolled for this study. The diagnosis of colorectal cancer was confirmed with colonoscopy and biopsy. Preoperative workup has included blood tests, chest X-ray, and serum carcinoembryonic antigen (CEA). CT scan was a routine. The surgical approach was decided with the consent of the patients after thorough discussion on the pros and cons of the approach. Patients with large, fixed tumors with invasion to other organs were excluded from laparoscopic trial. The patients have received mechanical bowel preparation the day before surgery and prophylactic intravenous antibiotics were administered at the time of induction of anesthesia. A urinary catheter was inserted after the patient was put under general anesthesia. Nasogastric tube was not used as a routine.

The patients were placed in a supine head down poison. At the beginning of the procedure, the peritoneal cavity was accessed through an insufflations' needle and carbon dioxide was insufflated to maintain the intra-abdominal pressure at 10–12 mm Hg. Three ports were used in all cases except one of total colectomy for which 5 ports have been used; however only three ports were used at a time.

**Figure 1:**
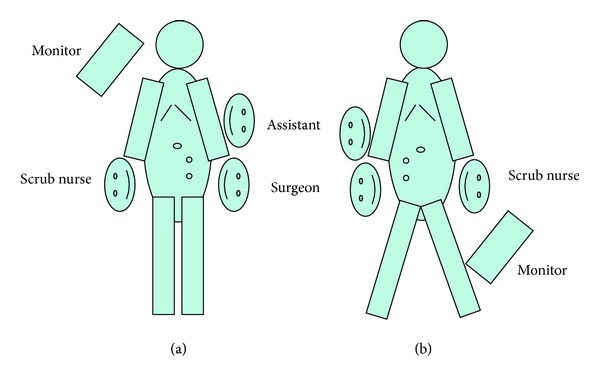
Patient positioning and ports distribution in 3 ports technique. (a) Right hemicolectomy and (b) rectosigmoid resection.

**Figure 2:**
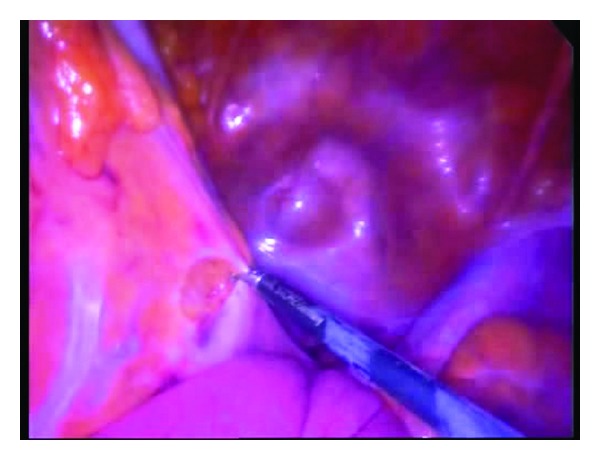
Medial to lateral rectosigmoid mobilization using 3 ports technique.

**Figure 3:**
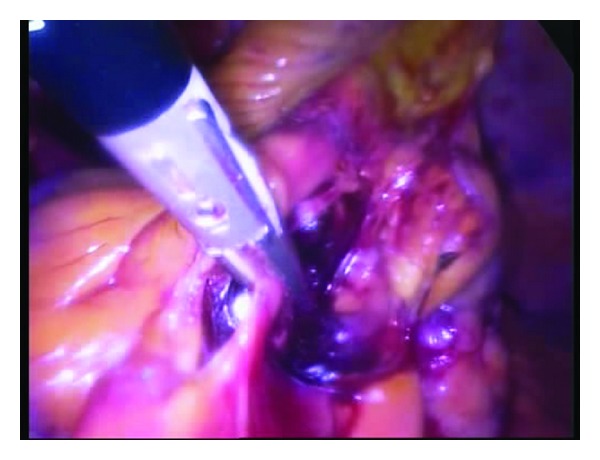
Medial approach for right hemicolectomy using 3 ports technique.

The ports were positioned so that convenient and safe dissection could be done. For right colon cancer, transumbilical 10 mm port was used for the camera and another 2 ports of either 5 or 10 mm size at the left mid clavicular line were placed as shown in Figure 1(a). For rectosigmoid cancer, the two ports were placed as in Figure 1(b). Dissection was performed in the majority of patients by alternate between monopolar and bipolar vascular sealing devices (Figures 2 and 3). Vessels were controlled with bipolar vascular sealing device or absorbable clips intracorporeally in most circumstances. Following bowel mobilization and vessel division, the tumor-bearing segment was retrieved through an incision (4-5 cm long) at a convenient site with adequate wound protection. In case of a right sided colonic lesion, resection and anastomosis were performed extracorporeally by hand sutures. Colorectal or coloanal anastomosis was performed by hand suturing or using a circular stapler which was inserted transanally. Colorectal mobilization and transaction followed the same principles as in open surgery.

Conversion was defined as the need for premature making of the abdominal incision for bowel mobilization and/or vascular control. Operative mortality was defined as death that occurred during the same hospital stay or within 30 days following the primary operation. Operative morbidities were defined as complications that result in prolonged hospital stay or additional interventions or procedures [8].

### 2.1. Data Collection and Statistical Analysis

Data on the patient's demographics, medical comorbidities, location of the tumors, operative details, postoperative outcomes, and follow-up status were collected prospectively and entered into a data base.

## 3. Results

In the period of June 2011 till December 2012, 24 cases (13 males and 11 females) of colon cancer have been enrolled for laparoscopic colorectal resection. The mean age was 47 years (39–72 ys).  Table 1 shows the demographic and clinical characteristics of the patients.

Right hemicolectomy was performed for 10 cases. Left hemicolectomy with sigmoidectomy was performed for 3 cases of proximal sigmoid cancer and anterior resection was done for 7 cases of rectosigmoid cancer. Total colectomy with ileorectal anastomosis was performed for one case of cecal cancer on top of familial adenomatous polyposis coli (FAP). Three cases (12.5%) have been converted because of local advancement (two cases) and bleeding (one case); see Table 2.

The mean operative time was 110 minutes (95–195). The mean estimated blood loss was 70 mL (40–90 mL). No major intraoperative complications have been encountered. The mean time for passing flatus after surgery was 36 hours (12–72 hours). The mean hospital stay was 5 days (4–7 days). The perioperative period passed without events.  Table 3 summarizes the perioperative results.

Pathologic outcome revealed that the mean number of retrieved lymph nodes was 14 (range 5–23 lymph node) and all cases have free surgical margin; see Table 3.

## 4. Discussion

The classic laparoscopic colorectal surgeries are performed through multiports allowing variation of scope and other instruments placement for easy and safe dissection. However in SILC, no additional ports exist and maneuvering is greatly restricted by nearby instruments. Therefore SILC requires an experienced surgeon to overcome the difficulties of triangulation, pneumoperitoneum leakage, and instruments crowding [10]. Additional ports have been recommended for the safe completion of SILC [11].

In a trial to overcome the difficulties of SILC, we have developed 3 ports technique which combines some advantages of conventional laparoscopy as well as SILC as a step for further refinement of reduced port surgery for colorectal cancer. In this series, we used 3 ports for laparoscopic colectomy. Positioning of the ports differs according to the surgical technique (right hemicolectomy, left hemicolectomy, or anterior resection). Surgeon was standing on the left when mobilizing and dissecting the right colon and on the right when operating on the left colon (Figure 1). For most of the cases, dissection and mobilization were performed in a medial to lateral fashion as in Figures 2 and 3; however, for the first 3 cases of right colon cancer, dissection and mobilization were performed in a lateral to medial fashion. Although we did not necessitate adding more ports in this series, it is possible to add other ports during the procedure if needed. We included cases of rectosigmoid cancers because some studies show that the pattern of recurrence and survival of patients with upper rectal cancer were similar to those of sigmoid cancer and technically anterior resection for upper rectal cancer does not differ significantly from surgery for a sigmoid cancer [12].

In this series of 3 ports laparoscopic colectomy, the patients have average body mass index (BMI) of 29 (26–33.5). Laparoscopic colectomy is safe and feasible in patients with high BMI, with no significant difference in recovery of intestinal function and length of hospital stay compared to patients with normal BMI [13, 14].

Operative morbidity is a very important issue; in this series the mean operative time was 110 minutes and mean estimated blood loss was 70 mL which are similar to other series [8].

In comparison to other studies, laparoscopic resection in our series has a similar short duration of ileus and an earlier resumption of diet. The hospital stay was also significantly short. Although we did not experience tremendous pressure for a short hospital stay and early discharge, the median hospital stay for our patients with laparoscopic resection was 5 days. Our series had very similar short-term results to that of North American and other large multicentre trials and the meta-analysis of randomized control trials. These trials have reported on the short-term outcomes and showed that significant early benefits measured as less surgical complications, less intraoperative blood loss, and less narcotic use can be achieved with laparoscopy. The meta-analysis also noted a significantly short time to first bowel movement and discharge from hospital [15–22].

Dissection through small incision, precise dissection helped by magnification, and avoidance of visceral handling are helping in early recovery of gut function [7, 16, 22]. There was no evidence of deep venous thrombosis or pulmonary complications except for one case with hypertension and diabetes. This is because of early ambulation and less postoperative pain. All these factors have contributed to the short postoperative hospital stay.

We believe that the most important thing in cancer surgery is to respect the oncologic principals. In this series, the mean number of retrieved lymph nodes was 14 which is consistent with the minimum of 12 lymph nodes required for accurate staging [22]. All cases have negative surgical margin. Therefore our pathological results were similar to those of most large trials [6, 7, 16, 22].

For further development of this technique, now we try to position the ports so that, at the end of dissection, two ports can be merged for specimen extraction and extracorporeal anastomosis. While SILC requires high volume surgeon, our target is to make RPS feasible by average laparoscopic colorectal surgeons with classical laparoscopy instruments.

In conclusion, three ports laparoscopy is a safe and effective procedure, has added advantages of cosmesis, and looks to have more advantages in short-term outcome than multiports laparoscopy. Pathological outcomes revealed that oncologic principals could be respected in 3 ports laparoscopy. Now, we are trying further refinement of reduced port surgery.

## Figures and Tables

**Table 1:** Patient's characteristics.

Variable	Average	Range
Age	47	39–72
Sex, M/F	13/11	
BMI	29	26–33.5

**Table 2:** Surgical procedures and lesions distribution.

Procedure	Lesion	Number	%
(1) Rt. hemicolectomy	Cecal	6/24	25
	Rt. colon	2/24	8.3
	Hepatic flexure	2/24	8.3
(2) Lt. Hemicolectomy with sigmoidectomy	Proximal sigmoid	3/24	12.5
(3) Anterior resection	Rectosigmoid	7/24	29.5
(4) Total colectomy	FAP with cecal cancer	1/24	4.2
(5) Conversion		3/24	12.5

**Table 3:** Operative and pathologic findings.

Variable	Mean	Range
Blood loss (mL)	70	45–90
Operative time (min.)	110	95–195
Conversion (no, %)	(3, 12.5%)	—
Time to passing gas (hours)	36	36–72
Time to oral fluid (days)	1.5	1.5–2.5
Time to oral semisolid (days)	3	2.5–4
Postoperative complications	0	—
Hospital stay (days)	5	4–7
Pathologic findings		
Retrieved LNs	14	5–23
Positive margin	0/21	—
